# Differences in the Gut Microbiota in Long-Term Infertile Holstein Repeat Breeder Cows and Healthy Fertile Holstein Cows

**DOI:** 10.3390/ani15182637

**Published:** 2025-09-09

**Authors:** Yui Kitagawa, Sayoko Oishi, Karen Koshimizu, Ryotaro Miura, Seizo Hamano, Hisataka Iwata, Koumei Shirasuna

**Affiliations:** 1 Department of Animal Science, Tokyo University of Agriculture, Atsugi 243-0034, Kanagawa, Japanh1iwata@nodai.ac.jp (H.I.); 2 Department of Large Animal Clinical Sciences, School of Veterinary Medicine, Rakuno Gakuen University, Ebetsu 069-0836, Hokkaido, Japan; r-miura@rakuno.ac.jp; 3 Artificial Insemination Association of Japan, Koto 135-0041, Tokyo, Japan; hamano@liaj.or.jp

**Keywords:** repeat breeder cows, gut microbiota, infertility, biological markers, metabolic pathways

## Abstract

Repeat breeder (RB) cows are subfertile animals and represent a major issue in the livestock industry. The influence of the gut microbiota on reproductive function and the gut microbiota–gonadal axis has garnered scientific attention. This study aimed to evaluate the relationship between reproductive performance and gut microbiota in Holstein cows, depending on reproductive attempts (number of artificial inseminations or embryo transfers) and subsequent pregnancy outcomes. Fecal samples were collected from Holstein cows at the time of ovulation and classified into four groups: Normal pregnancy (*n* = 27, ≤three reproductive attempts, subsequently fertile), Normal open (*n* = 25, ≤three reproductive attempts, subsequently infertile), RB pregnancy (*n* = 38, over four reproductive attempts, subsequently fertile), and RB open (*n* = 60, over four reproductive attempts, subsequently infertile). The PLS-DA analysis revealed differences in classification among groups, especially between Normal pregnancy and RB open. Regarding α- and β-diversity analyses, these indices were significantly different among the four groups (*p* < 0.05). Using LEfSe analysis, three genus-level bacteria (*Bacillus*, *Ruminococcus*, and *Sphingobium*) were identified as specific bacteria in the Normal pregnancy group, and eight genus-level bacteria (*Huintestinicola*, *Intestinimonas*, *Neglecta*, *Oscillibacter*, *Zongyangia*, *Phocaeicola*, *Alistipes*, and *Acholeplasma*) were identified as specific bacteria in both open groups (*p* < 0.05). KEGG analysis predicted some pathways (Apoptosis, Lipopolysaccharide biosynthesis, etc.) to be enriched in both the open groups. MetaCyc analysis predicted amine and polyamine degradation/biosynthesis as the specific changing pathways in the RB open group. This study demonstrated that the composition of the gut microbiota differs between normal fertility and repeated infertility conditions. Furthermore, even with fewer reproductive attempts, differences in gut microbiota composition were observed between cows that conceived and those that failed to conceive.

## Simple Summary

The influence of the gut microbiota on reproductive function has garnered scientific attention. This study investigated the relationship between reproductive performance and gut microbiota in Holstein cows, depending on reproductive attempts and subsequent pregnancy outcomes. Fecal samples were collected from Holstein cows at the time of ovulation and classified into four groups: normal pregnancy, normal open, repeat breeder (RB) pregnancy, and RB open. This microbiotal analysis suggested that the composition of the gut microbiota differs between normal fertility and repeated infertility conditions in Holstein cows. Furthermore, even with fewer reproductive attempts, differences in gut microbiota composition were observed between cows that conceived and those that failed to conceive.

## 1. Introduction

Cattle fertility is a major concern in the livestock industry. Repeat breeder (RB) cows are subfertile animals without anatomical or infectious abnormalities and do not become pregnant after three or more breeding attempts [1,2]. The presence of RB cows has been reported worldwide. Many studies have examined the factors that contribute to the repeat breeding syndrome (RBS), including subclinical endometritis, nutritional conditions, abnormal estrous behavior, abnormal hormonal regulation, and lower quality of oocyte and embryo [2,3,4,5]. Additionally, the uterine environment is associated with one of causing of RBS [2]. Kimura et al. [6] demonstrated that, even with the transfer of an elongating embryo, the pregnancy rates of low-fertility cows were dramatically lower than those of healthy fertile cows. Remarkably, the worse the condition of feces, the lower the conception rate after embryo transfer (ET), indicating the importance of the mother’s condition for pregnancy in cows [7].

The microbiome is well known to be important for the development of life in humans and animals [8]. It is not only greatly involved in various indispensable physiological activities but is also closely associated with the occurrence of multiple diseases [8]. An abnormal gut microbiome is frequently associated with a higher risk of intestinal permeability, which impairs the gut barrier and allows leakage of pathogens from the gut lumen into the body, resulting in an increase in inflammatory responses [8]. The microbiota in bovines is influenced by various factors, such as hormonal imbalance, pathogenic invasion, environmental stress, and antibiotic usage, which can adversely affect the microbiota and may lead to microbial dysbiosis associated with dysregulated reproductive function [9]. Recently, the influence of gut microbiota on reproductive function and the gut microbiota–gonadal axis has attracted attention [10]. Deng et al. [11] investigated the association between fecal microbial composition and reproductive performance after artificial insemination (AI) in beef heifers and found specific changes in the microbiota associated with pregnancy status, demonstrating that the bovine fecal microbiome could be used as a biomarker of bovine reproduction. Moreover, Taguchi et al. [12] reported that the fecal bacteriomes of Japanese black cattle heifers prior to AI were associated with AI attempts. However, the relationship between the gut microbiota and reproductive performance, especially in RB Holstein cows, has rarely been reported.

It has been reported that there is a relationship between changes in gut microbiota composition and female infertility in humans [13]. The purpose of the present study was to evaluate the relationship between reproductive performance and the gut microbiota in Holstein cows, where RBS is a major problem. The findings of this study allow insights into the prediction of reproductive performance in Holstein cows and for efficient breeding management in the future.

## 2. Materials and Methods

### 2.1. Design of Animal Experiments and Sampling

All Holstein cows (*n* = 150) included in the present study were reared on commercial farms in Hokkaido, Japan. The subjects were cows that had undergone AI or ET mainly during natural estrus after parturition. Individuals with a low breeding frequency (three or fewer) were defined as Normal, whereas those with a high breeding frequency (four or more) were defined as RB. In previous studies, RB cows were characterized by normal estrous behavior, anatomically normal uterus and ovaries as determined by observation using ultrasound diagnostics, and the inability to conceive after three or more reproductive attempts following normal estrous behavior [1,14]. After selecting the individual cows for the present study, ovulation was confirmed the day after estrus, and fecal samples were collected during rectal examination. All fecal samples (*n* = 150) were packed into tubes and stored at −20 °C until analysis. In addition, because a decline in the quality of oocytes and embryos has been reported in RB cows, we evaluated fertility using ET rather than AI [2]. ET was performed 6–7 days after estrus was detection, and pregnancy was determined via ultrasound diagnostics approximately 40 days after estrus. Considering the possibilities of conception, the following four groups were established for the present experiment: Group 1, Normal pregnancy (*n* = 27); individuals that had bred less than twice prior to the experiment and were fertile by the ET of the present study. Group 2, Normal open (*n* = 25); individuals that had bred less than twice prior to the experiment and did not conceive by the ET of the present study. Group 3, RB pregnancy (*n* = 38); individuals that had bred more than thrice prior to the experiment and were fertile by the ET of the present study. Group 4, RB open (*n* = 60); individuals that had bred more than thrice prior to the experiment and did not conceive by the ET of the present study. In addition, the average of number of breeding attempts and the average of number of parturitions were investigated, respectively.

### 2.2. DNA Extraction and 16S rRNA Sequencing

DNA was extracted from samples using E.Z.N.A. Stool DNA Kit (Omega Bio-tek, Norcross, GA, USA) according to the manual instructions from BGI (Hyogo, Japan). The sample (100–200 mg) was transferred to a centrifuge tube with grinding beads. After 1 mL ATL/PVP-10 buffer was added, the sample was ground in a grinding machine (Shanghai Jingxin Tech, Shanghai, China) and incubated at 65 °C for 20 min. The mixture was centrifuged at 14,000× *g* for 5 min (Eppendorf, Hamburg, Germany). The supernatant was transferred to a new tube. The PCI buffer (0.6 mL) was added to the samples and vortexed thoroughly for 15 s. The mixture was centrifuged at 18,213× *g* for 10 min. The supernatant was transferred to a deep-well plate with magnetic bead-binding solution (600 μL buffer with magnetic beads + 20 μL Proteinase K + 5 μL RNase A, 700 μL Wash 1, 700 μL Wash 2, 700 μL Wash 3, and 100 μL elution buffer). The sample was transferred to the corresponding location in the deep-well plate (Kingfisher; Thermo Fisher Scientific, Waltham, MA, USA). The corresponding program was initiated in the Kingfisher. The DNA was transferred to a 1.5 mL centrifuge tube for storage when the program runs out.

The library was prepared using 2 × Phanta Max Master Mix (VAZYME, Nanjing, China) polymerase, and the V3V4 variable region of bacterial 16S rDNA was amplified using forward and reverse PCR degenerate primers F and R (338F: ACTCCTACGGGAGGCAGCAG, 806R: GGACTACHVGGGTWTCTAAT). PCR enrichment was performed in 50 μL of reaction mix containing 30 ng template and fusion PCR primers. PCR cycling conditions were as follows: 95 °C for 3 min; 30 cycles of 95 °C for 15 s, 56 °C for 15 s, and 72 °C for 45 s; and final extension at 72 °C for 5 min. The PCR products were purified using DNA magnetic beads (LB00V60; BGI, Shenzhen, China).

Next, the final double-stranded library products were denatured to generate single-stranded library products. The circularization reaction was then performed to obtain single-stranded circularized DNA products. Single-stranded linear DNA was digested before removal. The final single-stranded circularized library was amplified with phi29 and rolling circle amplification to generate the DNA nanoball (DNB), which carried multiple copies of the initial single-stranded library molecule. The DNBs were loaded onto the patterned nanoarray, and sequencing reads of PE300 bases were generated using the DNBSEQ-G400 platform (BGI, Wuhan, China).

Fecal 16S rRNA sequencing was performed at BGI. Briefly, raw data were filtered to obtain high-quality clean data, after which clean reads that overlapped were merged into tags and further clustered into operational taxonomic units (OTUs). Taxonomic classifications were assigned to the representative OTU sequences using the Ribosomal Database Project database. Analyses of α-diversity, β-diversity, differential species, and network and model prediction were performed based on the OTU profile table and taxonomic annotation results.

### 2.3. Analysis of 16S rRNA Sequencing Data and Statistical Analysis

Subsequent analyses were performed using data files obtained from BGI. Principal component analysis (PCA), partial least squares discrimination analysis (PLS-DA), and Venn diagram analysis were performed based on the distribution of the OTUs among the samples. α-diversity indexes, such as the Shannon and Simpson indexes, were calculated and displayed as box plots. β-diversity analysis was conducted to investigate the structural variations in microbial communities across samples using Bray–Curtis metrics and weighted UniFrac, which were visualized via principal coordinate analysis (PCoA). Linear discriminant analysis effect size (LEfSe) was performed to identify statistically differentially abundant taxa between groups, and the values of linear discriminant analysis (LDA) > 2 and *p* < 0.05 were considered significantly different. Microbial functions were predicted through phylogenetic investigation of communities via reconstruction of unobserved states (PICRUSt2) based on Kyoto Encyclopedia of Genes and Genomes (KEGG) enrichment analysis and MetaCyc metabolic pathway analysis. Data were expressed as the mean ± standard error (SEM). Multiple comparisons were performed using a one-way analysis of variance, followed by Tukey’s multiple comparison test or the Kruskal–Wallis test followed by Steel–Dwass using statistical software (BellCurve version 4.08; Social Survey Research Information, Tokyo, Japan). Statistical significance was set at *p* < 0.05.

## 3. Results

### 3.1. Basic Information of Each Group Used in the Experiment

We divided the animals into four experimental groups according to reproductive performance: Normal pregnancy, Normal open, RB pregnancy, and RB open. In the present study, the average timing of fecal sampling was 124 ± 10 days in Normal pregnancy, 149 ± 7 days in Normal open, 222 ± 11 days in RB pregnancy, and 214 ± 8 days in RB open, after parturition, respectively. The average number of parturitions was 2.0 ± 0.2, 2.6 ± 0.4, 2.2 ± 0.2, and 2.5 ± 0.2 in Normal pregnancy, Normal open, RB pregnancy, and RB open, respectively. The average number of breeding attempts was 2.3 ± 0.2, 2.7 ± 0.1, 4.7 ± 0.2, and 4.7 ± 0.1 in Normal pregnancy, Normal open, RB pregnancy, and RB open, respectively.

### 3.2. Gut Microbiota Differs Depending on Breeding Attempts and the Circumstances of Subsequent Conception

**Figure 1:**
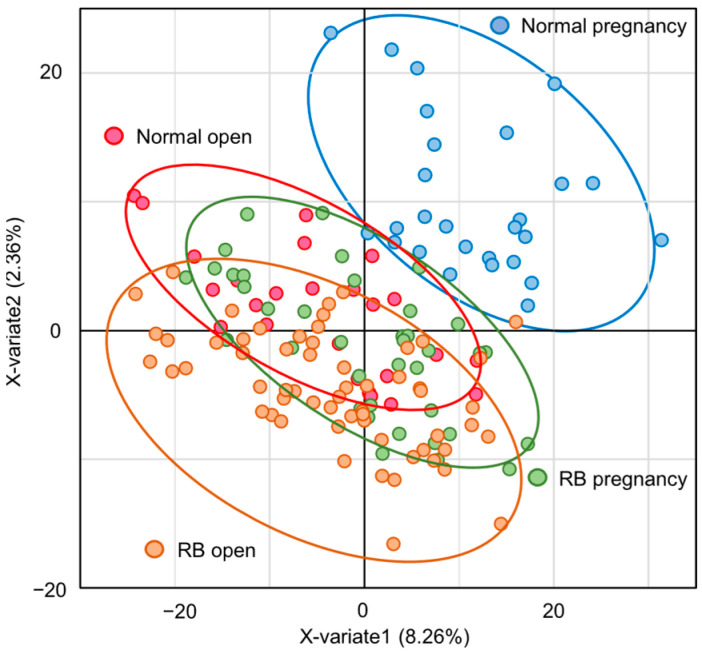
Differences in gut microbiota among groups according to PLS-DA analysis. PLS-DA analysis plot of the gut microbiota in Holstein cows. Blue-lined circle represents individuals in Normal pregnancy group. Red-lined circle represents individuals in Normal open group. Green-lined circle represents individuals in RB pregnancy group. Orange-lined circle represents individuals in RB open group.

The Venn diagram shows that 4121 species were detected in the fecal microbiota of 150 cows, of which 69.4% were common to all four groups (Supplementary Figure S1A). In contrast, some bacteria were found only in one group (Supplementary Figure S1A). The results of the PCA showed no clear differences among the four groups (Supplementary Figure S1B). However, the results of PLS-DA analysis showed differences in classification among the four groups (Figure 1). In particular, while the classifications between the groups of Normal pregnancy and RB open were completely different, there was a large overlap between the Normal open and RB pregnancy groups (Figure 1).

### 3.3. Diversity of the Gut Microbiota Varies Depending on the Breeding Attempts and Circumstances of Subsequent Conception

**Figure 2:**
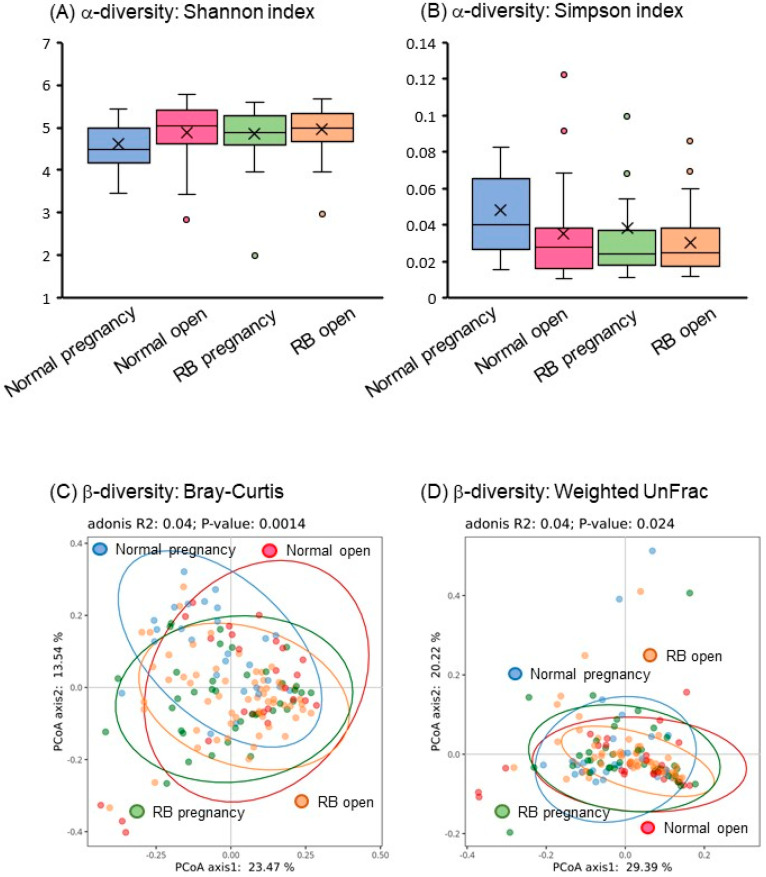
Gut microbial diversity in Holstein cows. (**A**,**B**) Shannon and Simpson indexes as α-diversity of the fecal bacteriome of Holstein cows. (**C**,**D**) Bray–Curtis and weighted UniFrac analyses as β-diversity of the fecal bacteriome of Holstein cows. Blue-lined circle represents individuals in Normal pregnancy group. Red-lined circle represents individuals in Normal open group. Green-lined circle represents individuals in RB pregnancy group. Orange-lined circle represents individuals in RB open group.

In α-diversity analysis, the Shannon and Simpson indexes reflected the species diversity of the community, which was affected by species richness and evenness. Species diversity is proportional to the Shannon index values but is negatively correlated with the Simpson index. In the present study, both Shannon and Simpson indexes of the gut microbiota were significantly different (*p* < 0.05) among the four groups, especially in the Normal pregnancy group (Figure 2A,B).

In β-diversity analysis, the Bray–Curtis distance is a commonly used index that reflects the differences between two communities. UniFrac uses system evolutionary information to compare the composition of community species between samples. In this study, the PCoA of β-diversity based on Bray–Curtis distance and weighted UniFrac showed significant separation (*p* < 0.05) of fecal microbial communities among the four groups (Figure 2C,D).

These findings suggest that a significant difference exists in the gut microbial composition profiles of Holstein cows, depending on the number of breeding attempts and the circumstances of subsequent conception.

### 3.4. Relative Abundance of the Gut Microbiota Depends on Breeding Attempts and the Circumstances of Subsequent Conception

**Figure 3:**
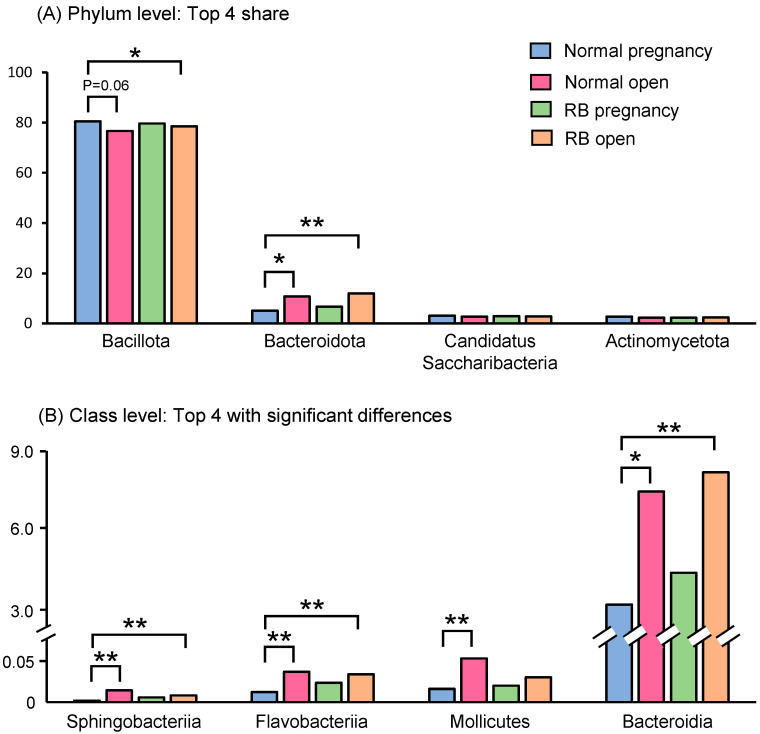
Relative abundances of phylum- and class-level bacteria in the feces of Holstein cows. (**A**) Relative abundances of top four shared bacteria at phylum level in the feces of Holstein cows. (**B**) Relative abundances of top four bacteria at class level with significant differences in the feces of Holstein cows. Asterisks indicate significant differences between groups (*: *p* < 0.05, **: *p* < 0.01).

At phylum level, the top four predominant bacteria in the feces of cows in the four groups were *Bacillota* (76–81%), *Bacteroidota* (5–12%), *Candidatus Saccharibacteria* (2.7–3.1%), and *Actinomycetota* (2.3–2.8%), the sum of their abundance accounted for 91.4–95.6% of the overall abundance (Figure 3A).

At the class level, the top three predominant bacteria in the feces of cows in the four groups were *Clostrida* (73–76%), unclassified (8.8–9.3%), and *Bacteroidia* (3.6–8.5%). We selected the top four significantly (*p* < 0.05) altered bacteria and the bacteria of classes *Sphingobacteriia*, *Flavobacteriia*, and *Bacteroidia* were found to be significantly (*p* < 0.05) enriched in the gut microbiota of cows in both open groups (Normal and RB) compared to the Normal pregnancy group (Figure 3B). Bacteria of the class *Mollicutes* were also present at higher levels in the Normal open group than in the Normal pregnancy group (Figure 3B).

**Figure 4:**
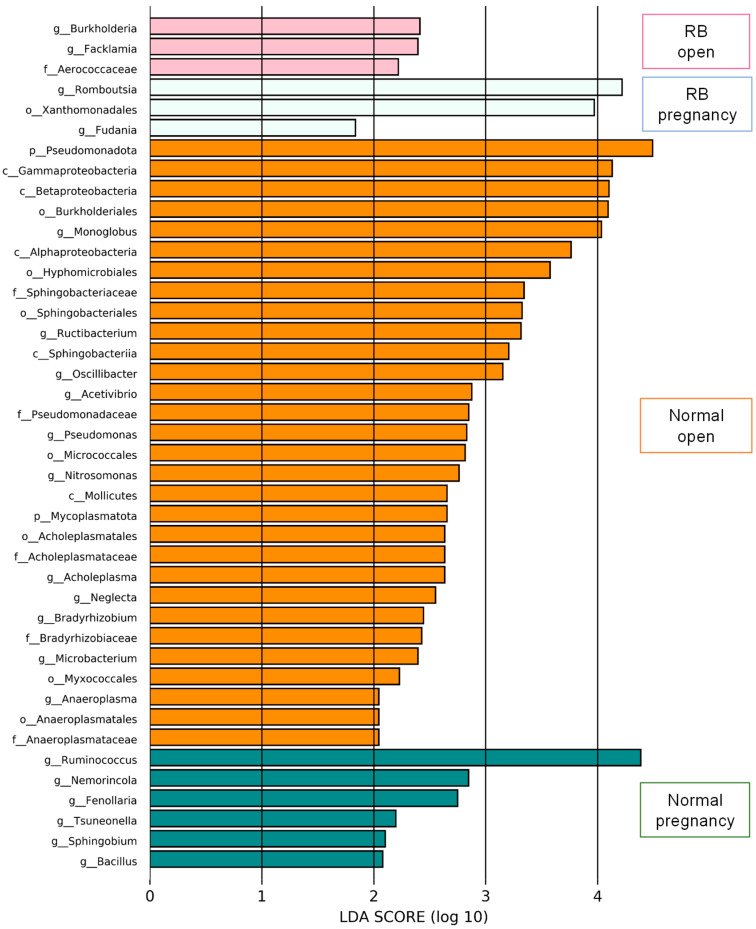
LEfSe screening for potential relationships among factors in the feces of Holstein cows. LEfSe score histogram of differential gut microbes between groups. Green bars indicate the Normal pregnancy group. Orange bars indicate the Normal open group. Light blue bars indicate the RB pregnancy group. Pink bars indicate the RB open group.

**Figure 5:**
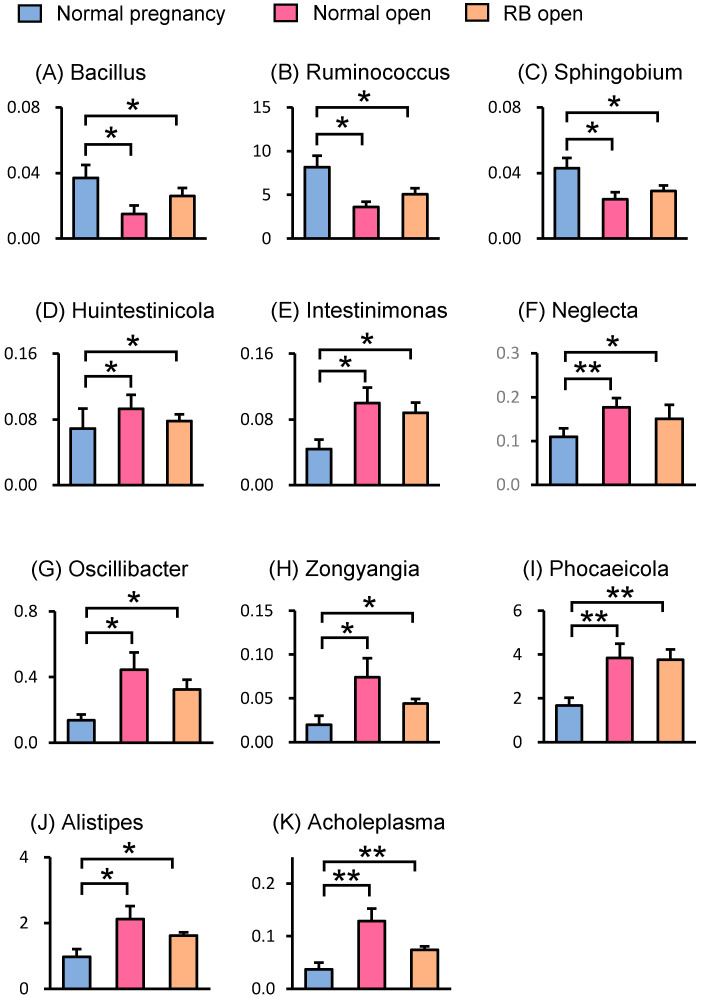
Relative abundances of genus-level bacteria in the feces of Holstein cows. Relative abundances of genus-level bacteria in the feces from LEfSe analysis are shown. (**A**–**C**) Three genera of bacteria were identified as characteristic bacteria that were more abundant in the Normal pregnancy group compared to the both open groups (Normal and RB). (**D**–**K**) Eight genera of bacteria were identified as characteristic bacteria that were less abundant in the Normal pregnancy group compared to those in the both open groups (Normal and RB). Data are presented as the mean ± SEM. Asterisks indicate significant differences between the group (* *p* < 0.05, ** *p* < 0.01).

LEfSe analysis was performed to identify the representative differential bacteria among the four groups, and the characteristic bacteria were identified in each of the four groups. For example, the bacteria of the genus *Ruminococcus* in the Normal pregnancy group and the bacteria of the phylum *Pseudomonadota* in the Normal open group were the most enriched (Figure 4). To further identify the characteristic bacteria related to conception, LEfSe analysis was performed between the groups of Normal open or RB open and Normal pregnancy (Supplementary Figures S2 and S3). Among the listed bacteria at the genus level, those that were common and detected in more than half of the individuals in each group were selected (Figure 5). Three genus-level bacteria, *Bacillus*, *Ruminococcus*, and *Sphingobium*, were found to be significantly (*p* < 0.05) more abundant in the Normal pregnancy group than in the both open groups (Normal and RB) (Figure 5A–C). On the other hand, eight genus-level bacteria, namely *Huintestinicola*, *Intestinimonas*, *Neglecta*, *Oscillibacter*, *Zongyangia*, *Phocaeicola*, *Alistipes*, and *Acholeplasma*, were significantly (*p* < 0.05) more abundant in the both open groups (Normal and RB) than in the Normal pregnancy group (Figure 5D–K).

We listed the genus-level bacteria that varied in the open groups, both Normal and RB, compared with the Normal pregnancy group (Supplementary Table S1). Bacteria identified as common variants in the LEfSe analysis (Figure 5) were similarly listed. In contrast, bacteria that showed significant variations when compared with the RB open group were also listed. For example, bacteria of the genus *Streptococcus* and *Terrisporobacter* showed a significant increase in abundance in the RB open group only when comparing the RB open with Normal pregnancy group, which was different from the change observed when the Normal open and Normal pregnancy groups were compared (*p* < 0.05).

Finally, we listed the top ten species-level bacteria with the highest occupancy rates (Table 1). When comparing each open group (Normal and RB) with the Normal pregnancy group, the species-level bacteria that showed significant variation in both groups were *Monoglobus pectinilyticus* and *Ruminococcus bovis* (*p* < 0.05). The abundance of *Bifidobacterium pseudolongum* was higher in the Normal pregnancy group than in the Normal (*p* < 0.1) and RB (*p* < 0.05) open groups.

These results suggest that, compared with the Normal pregnancy group, gut microbiota that differed at the phylum, class, genus, and species levels were formed in both open groups (Normal and RB) in Holstein cows.

### 3.5. Differences in the Gut Microbiota Function Between Normal Pregnancy and Both Open Groups (Normal and RB)

The predicted function of the gut microbiota in Normal pregnancy and both open groups (Normal and RB) in Holstein cows was inferred using PICRUSt2. Based on the KEGG pathway analysis using PICRUSt2, the top eight commonly changed predicted pathways are shown in Table 2. The pathways that showed the greatest variation were apoptosis, biosynthesis of siderophore group non-ribosomal peptides, geraniol degradation, and lipopolysaccharide biosynthesis, and the ratios were lower in both open groups (Normal and RB).

Based on the MetaCyc metabolic pathway analysis using PICRUSt2, the top five commonly changed predicted pathways are shown in the upper panel of Table 3. The pathways that showed the greatest variation were electron transfer, fatty acid and lipid degradation, and secondary metabolite degradation, and their ratios were lower in both open groups (Normal and RB). The pathway changes observed only in the comparison between Normal pregnancy and RB open groups are shown in the under panel of Table 3. Remarkably, both amine and polyamine degradation/biosynthesis were predicted as specific changing pathways in the RB open group compared to the Normal pregnancy group.

These results suggest that, compared to the Normal pregnancy group, the predicted function of the gut microbiota differs in both the open groups (Normal and RB).

## 4. Discussion

In the present study, PLS-DA revealed significant differences in classification, especially between the Normal pregnancy and RB open groups. In addition, α- and β-diversity also changed significantly among the four different groups. These findings suggest that the composition of the gut microbiota in Holstein cows varies according to the postpartum reproductive status and subsequent fertility. It is well known that gut microbiome diversity is important in healthy conditions, and that α-diversity is low in various diseases [15,16,17]. However, in the present study, α-diversity was lower in the good reproductive performance group (Normal pregnancy) in Holstein cows. Similarly, in the human infertility patient group, the α-diversity (Chao 1) index has shown an increasing trend compared to that in the control fertile subjects [18]. Therefore, it is possible that the high diversity is caused by the presence of the abnormal gut microbiota that is linked to infertility.

Next, we verified changes in the gut microbiota of Holstein cows by comparing bacterial abundance at the phylum and class levels. No effect due to the number of breeding attempts up to that point (Normal vs. RB groups) was observed. The prevalence of the phylum *Bacteroidota* and classes *Bacteroidia*, *Sphingobacteriia*, and *Flavobacteriia* was high in both groups that subsequently became infertile (Normal open and RB open). Therefore, we suggest that the gut microbiota composition in Holstein cows differs significantly between individuals who subsequently conceive and those who do not, rather than between the Normal (few breeding attempts) and RB (many breeding attempts) groups. In a human study, no significant difference was found in the abundance of the phyla *Bacillota* (*Firmicutes*) and *Bacteroidota* (*Bacteroidetes*) between control subjects and patients with infertility. However, the abundance of the phylum *Verrucomicrobia* was higher in the infertile patient group than in the control group [18]. In a study involving Japanese Black heifers, the family *Erysipelotrichaceae* was strongly associated with an increase in the AI number [12]. In contrast, the orders *Bacteroidales* and *Lachnospiraceae* were more abundant in the feces of beef heifers that became pregnant after breeding [11]. Although the gut microbiota may be useful for predicting subsequent pregnancy, it is highly likely that the characteristic bacteria will differ depending on the species and sampling time.

To identify the bacteria that have a positive effect on conception, we combined the variation rates of LEfSe and bacterial genus levels and identified three bacterial species that were more abundant in the Normal pregnancy group (Figure 5A–C). Species-level comparisons revealed that *Ruminococcus bovis*, which plays a potential role in improving metabolic disorders [19], was more prevalent in the Normal pregnancy group. Among them, the genera *Bacillus* and *Ruminococcus* were the groups of bacteria that produced short-chain fatty acids (SCFAs) [20,21]. Short-chain fatty acids exert anti-inflammatory effects and induce regulatory T cells, which are important for the establishment of pregnancy [22,23,24]. Indeed, *Bacillus coagulans* can improve inflammatory conditions by regulating anti-inflammatory cytokines and T cells [20]. In humans, probiotics such as *Bacillus subtilis* can prevent recurrent spontaneous preterm deliveries [25]. In addition, a higher abundance of *Ruminococcus bromii* is positively associated with fecal butyrate levels and decreased inflammatory disease [26]. Notably, *Bifidobacterium pseudolongum* was found to be more abundant in the Normal pregnancy group than in the both open groups (Normal and RB) in Holstein cows. Recently, Song et al. [27] reported that *Bifidobacterium pseudolongum* produces acetate (an SCFA) and inhibits inflammatory signaling, resulting in a healthy gut microbiome composition and improved gut barrier function. Therefore, we hypothesized that one possible reason for good reproductive performance of the Normal pregnancy group in the present study was the greater presence of SCFA-producing bacteria.

Additionally, the genus *Sphingobium* was identified to be positively associated with pregnancy in the present study. Certain type of bacteria in the genus *Sphingobium* can synthesize polyamines, including spermidine [28]. Polyamines play multiple essential roles in reproductive functions such as embryo development, implantation, placental development, and interferon tau production and function [29,30]. Supplementation with spermidine improves oocyte quality in aged mice, leading to increased fertility [31]. In addition, supplementation with spermidine improves oocyte and embryo quality by regulating mitochondrial function and oxidative stress damage in mice and pigs [32,33]. In the present study, the RB open group was predicted to have overactive polyamine synthesis and degradation systems due to the influence of gut bacterial dysbiosis, and we speculated that abnormalities in polyamine biosynthesis are associated with RBS in Holstein cows. In the future, it will be necessary to verify polyamine concentrations in RB and/or infertile cows and their effects on the reproductive function of Holstein cows.

In contrast to the Normal pregnancy group, combining the variation rates of LEfSe and genus levels of bacteria, we identified eight bacteria that are more abundant in both open groups (Normal and RB; Figure 5). Similar to the present results, the genus *Alistipes* was more abundant in patients with infertility than in control subjects [18]. The relative abundance of the genus *Intestinimonas* was higher in maternal fecal samples from mice fed a high-fat diet compared with that in mice fed a normal diet [34]. Bacteria belonging to the genus *Acholeplasma*, a member of the class *Mollicutes*, are pathogenic to bovine oviduct cells [35]. Therefore, the gut microbiota of Holstein cows that subsequently become infertile might possess a high number of bacteria that could worsen the condition.

Many characteristic bacteria were detected in the open groups, and their predicted functions were examined using PICRSt2 analysis. In KEGG pathway analysis, geraniol degradation was the most significant pathway that changed, with an increase in the gut microbiota of the Normal open group. Because geraniol has anti-inflammatory effects [36], its degradation may be associated with inflammation in infertile Holstein cows. The biosynthesis pathway of siderophore group non-ribosomal peptides was higher in the gut microbiota of the both open groups, and this pathway is enriched in human patients with iron deficiency anemia [37]. Moreover, a higher occurrence of apoptosis and lipopolysaccharide (LPS) biosynthesis pathways were predicted in the gut microbiota in both infertility groups in the present study. Although LPS is produced by gut bacteria even under healthy conditions, it can induce stronger inflammatory responses in diseased states [38,39]. In addition to local effects, bacterial LPS crosses the gut barrier and enters the circulation systemically to induce inflammatory responses [40]. LPS is an important risk factor for reduced reproductive performance in Holstein cows [41,42]. We recently reported that the uterine microenvironment in RB cows was inflammatory, and that LPS levels in the uterine fluid were higher in the RB group than in the normal group [43]. Yagisawa et al. [44] reported that bacterial microbiota exists in the uterus of dairy cows and differs between fertile and infertile cows. These data suggest that gut bacteria or bacteria-derived LPS in cows are transmitted into the uterus and are involved in reduced fertility.

The limitations of this study are as follows. (1) In this study, the gut microbiota in Holstein cows was analyzed to verify its correlation with fertility; therefore, in the future, metabolic products, inflammatory parameters, and hormone concentrations and their effects must be examined. (2) It will also be necessary to investigate how the identified bacteria and their metabolic products directly affect fertility in cows. Recently, Chen et al. [45] demonstrated the detailed role of *Clostridium innocuum*, an opportunistic gut pathogen in women with infertility, that *Clostridium innocuum* converts progesterone into the neurosteroid epipregnanolone, reduces plasma progesterone levels, further induces luteal phase insufficiency, and arrests ovarian follicular development in female mice. Indeed, progesterone levels during the estrous cycle tend to be lower in repeat-breeding dairy heifers than in healthy heifers [3]. (3) The gut microbiota is affected by nutrition such as feed and the rearing environment, so analysis must also take these points into consideration. We plan to conduct more detailed studies on the above points in the future. Further research on these topics may lead to the development of new breeding enhancement techniques that utilize microbiota or metabolic products.

## 5. Conclusions

The present study demonstrated that the composition of the gut microbiota differs between normal fertility and repeated infertility conditions. Furthermore, even when reproductive attempts were few, the composition of the gut microbiota differed between individuals who subsequently conceived and those who did not. The results of the present study suggest that the microbiota may be able to predict fertility before reproductive attempts in Holstein cows.

## Figures and Tables

**Table 1:** List of the gut bacteria by species in order of prevalence.

Species Top 10	Normal Pregnancy	Normal Open	RB Open	Ratio (Normal Pregnancy/Normal Open)	*p* Value	Ratio (Normal Pregnancy/RB Open)	*p* Value
Romboutsia_timonensis	3.670	2.979	3.721	1.23	0.183	0.99	0.366
Paeniclostridium_sordellii	2.661	2.365	3.314	1.13	0.501	0.80	0.111
Monoglobus_pectinilyticus	1.104	1.679	1.409	0.66	0.008	0.78	0.047
Bifidobacterium_pseudolongum	0.867	0.108	0.104	8.06	0.079	8.33	0.033
Kineothrix_alysoides	0.696	0.806	0.773	0.86	0.637	0.90	0.766
Ruminococcus_bovis	0.556	0.325	0.397	1.71	0.001	1.40	0.015
Turicibacter_bilis	0.535	0.364	0.587	1.47	0.353	0.91	0.165
Lentihominibacter_hominis	0.490	0.494	0.568	0.99	0.513	0.86	0.554
Saccharofermentans_acetigenes	0.385	0.240	0.300	1.60	0.082	1.28	0.167
Ihubacter_massiliensis	0.381	0.551	0.496	0.69	0.082	0.77	0.005

**Table 2:** List of KEGG pathways ranked in order of significance based on normal group comparison analysis.

Changing KEGG Pathways: Normal Pregnancy vs. Open (Normal and RB)	Ratio (Normal Pregnancy/Normal Open)	*p* Value	Ratio (Normal Pregnancy/RB Open)	*p* Value
Geraniol degradation	0.49	0.0000	0.59	0.0020
Metabolism of xenobiotics by cytochrome P450	0.63	0.0000	0.72	0.0010
Flavonoid biosynthesis	0.69	0.0001	0.89	0.1348
Biosynthesis of siderophore group nonribosomal peptides	0.38	0.0002	0.47	0.0007
Apoptosis	0.33	0.0004	0.51	0.0036
Glyoxylate and dicarboxylate metabolism	0.97	0.0005	0.98	0.2206
Mismatch repair	1.02	0.0005	1.01	0.0394
Retinol metabolism	0.60	0.0006	0.57	0.0001
Lipopolysaccharide biosynthesis	0.52	0.0007	0.64	0.0017
Peptidoglycan biosynthesis	1.03	0.0008	1.03	0.0003

**Table 3:** List of MetaCyc metabolic pathways.

**Commonly Changing MetaCyc Metabolic Pathways Top 5:** **Normal Pregnancy vs. Open (Normal and RB)**	**Ratio (Normal Pregnancy/Normal Open)**	***p* Value**	**Ratio (Normal Pregnancy/RB Open)**	***p* Value**
Electron Transfer	0.57	0.000	0.68	0.002
Fatty Acid and Lipid Degradation	0.43	0.000	0.50	0.003
Amino Acid Biosynthesis	1.04	0.001	1.05	0.000
Secondary Metabolite Degradation	0.70	0.001	0.71	0.001
Aromatic Compound Biosynthesis	1.04	0.002	1.05	0.000
**Differentially Changing MetaCyc Metabolic Pathways:** **Normal Pregnancy vs. Open (Normal and RB)**	**Ratio (Normal Pregnancy/Normal Open)**	***p* Value**	**Ratio (Normal Pregnancy/RB Open)**	***p* Value**
Nucleoside and Nucleotide Degradation	0.99	0.243	0.91	0.000
Amine and Polyamine Degradation	1.05	0.611	0.85	0.024
Amine and Polyamine Biosynthesis	0.96	0.424	0.82	0.033

## Data Availability

The data presented in the present study are available upon request from the corresponding author for a clear reason.

## Supplementary Materials

The following supporting information can be downloaded at: https://www.mdpi.com/article/10.3390/ani15182637/s1, Figure S1: Differences in gut microbiota among groups according to Venn diagram (A) and PCA analysis (B). Figure S2: LEfSe screening for potential relationships among factors in the feces of Holstein cows between Normal pregnancy and Normal open groups. Figure S3: LEfSe screening for potential relationships among factors in the feces of Holstein cows between Normal pregnancy and RB open groups. Table S1: List of the gut bacteria by genus in order of significant difference.

## Abbreviations

The following abbreviations are used in this manuscript:

RB	Repeat breeder
RBS	Repeat Breeding Syndrome
ET	Embryo transfer
AI	Artificial insemination
DNB	DNA nanoball
OTU	Operational taxonomic unit
PCA	Principal component analysis
PLS-DA	Partial least squares discrimination analysis
PCoA	Principal coordinate analysis
LEfSe	Linear discriminant analysis effect size
SCFAs	Short-chain fatty acids
LPS	Lipopolysaccharide
