# Supplementary material for: Differences in the Gut Microbiota in Long-Term Infertile Holstein Repeat Breeder Cows and Healthy Fertile Holstein Cows
Source: Animals (Basel). 2025 Sep 9;15(18):2637. doi: 10.3390/ani15182637 (PMC12466702; doi:10.3390/ani15182637)
Supplement: Supplementary file 1 [file animals-15-02637-s001.zip › animals-3826855-supplementary.pdf]

animals

MDPI

Supplementary Figure 1

(A) Venn diagram

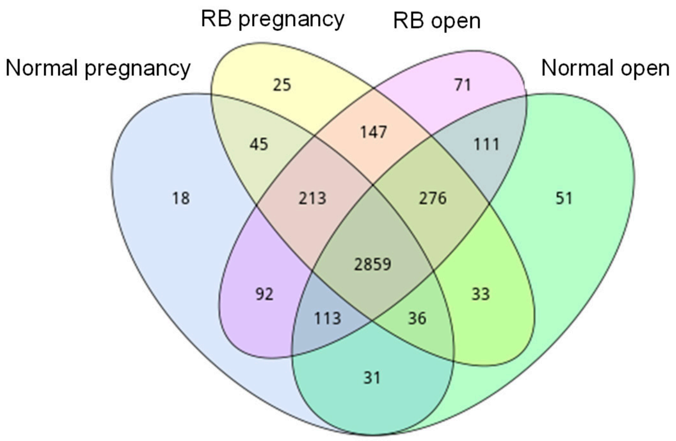

(B) PCA analysis

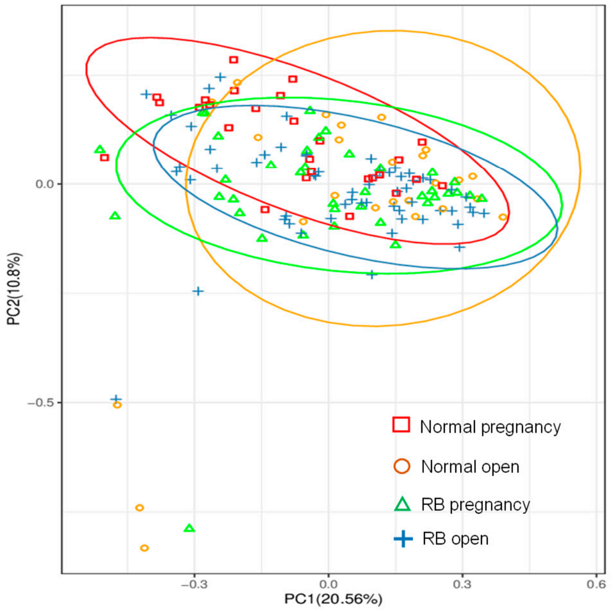

Supplementary Figure 1: Differences in gut microbiota among groups according to Venn diagram (A) and PCA analysis (B)

Figure S1A: Venn diagram of the gut microbiota distribution between groups.

Figure S1B**.** Differences in gut microbiota among groups according to PCA analysis. Red-lined square represents individuals in Normal pregnancy group. Orange-lined circle represents individuals in Normal open group. Green-lined triangle represents individuals in RB pregnancy group. Blue-lined cross represents individuals in RB open group.

Supplementary Figure 2

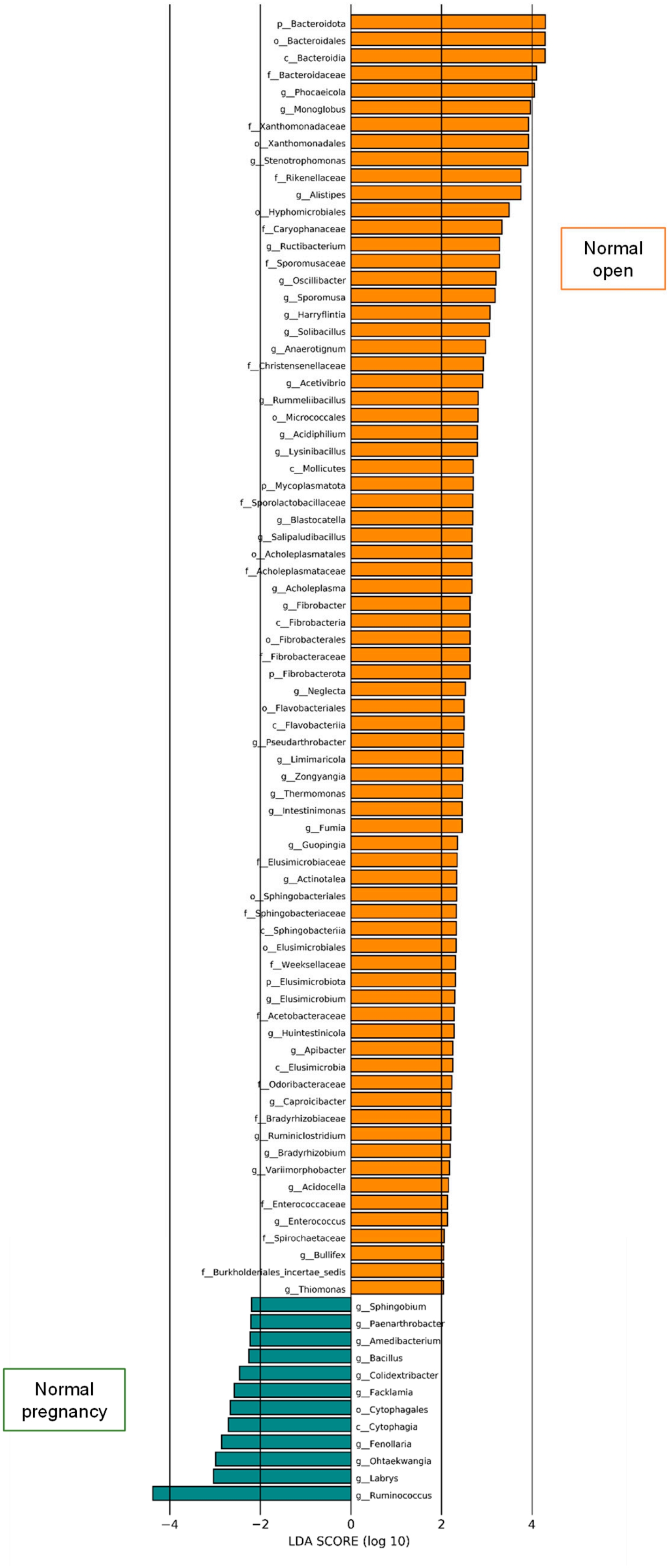

Supplementary Figure 2: LEfSe screening for potential relationships among factors in the feces of Holstein cows between Normal pregnancy and Normal open groups. LEfSe score histogram of differential gut microbes between groups. Green bars indicate a Normal pregnancy. Orange bars indicate the Normal open group.

Supplementary Figure 3

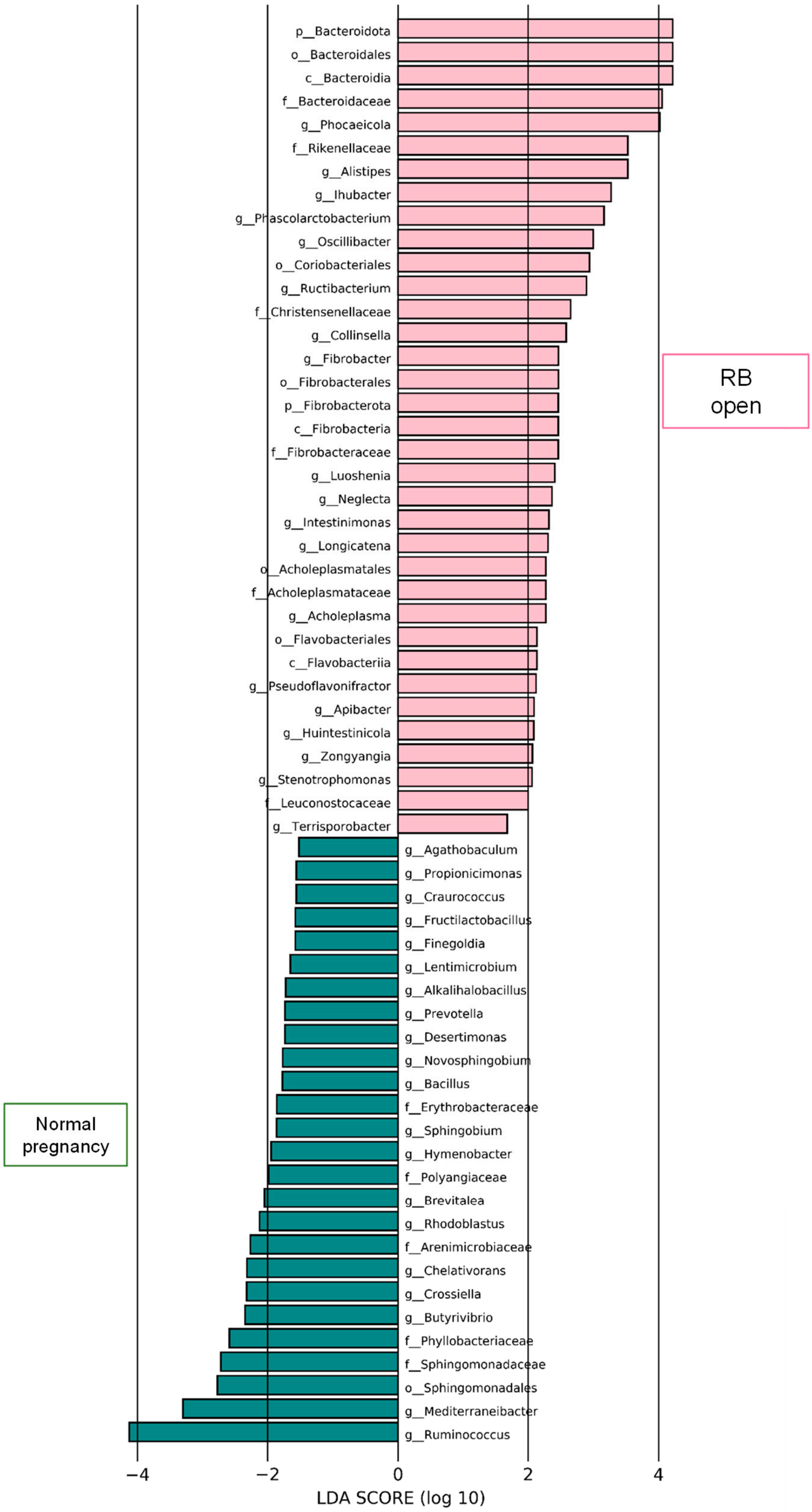

Supplementary Figure 3: LEfSe screening for potential relationships among factors in the feces of Holstein cows between Normal pregnancy and RB open groups. LEfSe score histogram of differential gut microbes between groups. Green bars indicate the Normal pregnancy. Pink bars indicate the RB open group.

Supplementary Table 1: List of the gut bacteria by genus in order of significant difference

Genus	Ratio (Normal pregnancy/Normal open)	p.value	Ratio (Normal pregnancy/RB open)	p.value
Acholeplasma	0.12	0.000	0.25	0.005
Acetivibrio	0.04	0.000	0.23	0.069
Ruminococcus	1.93	0.000	1.24	0.024
Ructibacterium	0.18	0.001	0.34	0.005
Colidextribacter	1.75	0.001	1.25	0.188
Bacillus	6.74	0.005	6.83	0.072
Phocaeicola	0.43	0.006	0.36	0.002
Sphingobium	2.12	0.007	1.52	0.032
Monoglobus	0.66	0.008	0.78	0.047
Oscillibacter	0.23	0.012	0.32	0.006
Zongyangia	0.18	0.014	0.21	0.021
Alistipes	0.24	0.021	0.34	0.007
Neglecta	0.57	0.022	0.54	0.010
Intestinimonas	0.42	0.023	0.39	0.008
Adlercreutzia	1.41	0.033	1.29	0.201
Huintestinicola	0.36	0.040	0.44	0.031
Enterococcus	0.40	0.044	0.66	0.194
Alloprevotella	0.20	0.045	0.24	0.040
Mediterraneibacter	1.29	0.047	1.24	0.033
Guopingia	0.26	0.054	0.28	0.044
Odoribacter	0.18	0.057	0.39	0.036
Pseudoflavonifractor	0.30	0.061	0.34	0.007
Ihubacter	0.69	0.082	0.77	0.005
Phascolarctobacterium	0.33	0.089	0.21	0.010
Anaerobutyricum	0.68	0.096	0.57	0.041
Pantoea	0.81	0.105	0.76	0.017
Luoshenia	0.27	0.131	0.23	0.024
Gehongia	0.52	0.138	0.47	0.037
Pseudobutyrivibrio	1.39	0.145	1.17	0.013
Anthropogastromicrobium	0.83	0.178	0.43	0.026
Streptococcus	0.91	0.222	0.45	0.045
Paraprevotella	0.28	0.236	0.36	0.050
Laedolimicola	0.47	0.373	0.49	0.036
Longicatena	0.41	0.379	0.23	0.019
Collinsella	0.69	0.458	0.59	0.034
Terrisporobacter	1.33	0.539	0.70	0.043
